# *MicroRNA*-*382* inhibits cell growth and migration in colorectal cancer by targeting SP1

**DOI:** 10.1186/s40659-018-0200-9

**Published:** 2018-11-24

**Authors:** Yupeng Ren, Hao Zhang, Peng Jiang

**Affiliations:** Department of Colorectal Surgery, Cancer Hospital of China Medical University, Liaoning Cancer Hospital & Institute, NO.44 Xiaoheyan Road, Dadong District, Shenyang, 110042 People’s Republic of China

**Keywords:** *MIR*-*382*, SP1, Colorectal cancer, Tumor progression

## Abstract

**Background:**

Emerging evidence showed that *microRNAs* (*miRs*) play critical roles in human cancers by functioning as either tumor suppressor or oncogene. *MIR*-*382* was found to function as tumor suppressor in certain cancers. However, the role of *MIR*-*382* in colorectal cancer (CRC) is largely unknown. Specificity protein 1 (SP1) is highly expressed in several cancers including CRC and is correlated with poor prognosis, but it is unclear whether or not *MIR*-*382* can regulate the expression of SP1.

**Methods:**

*MIR*-*382* expression level was measured by reverse transcription-quantitative polymerase chain reaction. The connection between *MIR*-*382* and SP1 was validated by luciferase activity reporter assay and western blot assay. Cell counting kit-8 assay and wound-healing assay were conducted to investigate the biological functions of *MIR*-*382* in CRC.

**Results:**

In this study, we found *MIR*-*382* expression was downregulated in CRC tissues and cell lines, and the transfection of *MIR*-*382* mimic decreased cell growth and migration. Furthermore, we identified SP1 was a direct target of *MIR*-*382*. Overexpression of *MIR*-*382* decreased the expression of SP1, whereas *MIR*-*382* knockdown promoted SP1 expression. We also observed an inversely correlation between *MIR*-*382* and SP1 in CRC tissues. Additionally, we showed that knockdown of SP1 inhibited cell growth and migration and attenuated the effect of *MIR*-*382* inhibitor on cell behaviors.

**Conclusions:**

In conclusion, the present study describes a potential mechanism underlying a *MIR*-*382*/SP1 link contributing to CRC development. Thus, *MIR*-*382* may be able to be developed as a novel treatment target for CRC.

## Background

Colorectal cancer (CRC) ranks as the third most commonly diagnosed cancer type worldwide [1, 2]. Owing to the improvements in therapeutic measures, the incidence rate of CRC has been declined in a worldwide range [3]. However, the CRC incidence and cancer-related death are rapidly increasing in Asian countries [4].

Specificity protein 1 (SP1), a transcription factor, has been demonstrated to play a critical role in the regulation of tumor-associated genes required for tumor survival, progression and metastasis [5]. In most cases, SP1 regulates gene expression mainly through GC-rich region binding but it is also capable to bind GT-rich sequences [6]. Elevated expression of SP1 was identified in several human cancers. For example, SP1 was overexpression in breast cancer and could regulate TINCR-*MIR*-*7*-KLF4 axis, which in turn stimulate cell proliferation but suppress cell apoptosis [7]. In ovarian cancer, upregulation of SP1 enhanced VEGF expression and resulted in angiogenesis and migration stimulation in vitro and in vivo [8]. Another study investigated the role of SP1 in hepatocellular carcinoma demonstrated that SP1 regulate cystathionine γ-lyase gene expression in human hepatocellular carcinoma cell lines to promote cell proliferation and cell cycle progression [9]. Moreover, SP1 is also found highly expressed in CRC and correlated with tumor metastasis and poor prognosis [10]. Accumulating evidence has demonstrated SP1 is a mediator for various cell behaviors including cell cycle, proliferation, and apoptosis [5, 11, 12]. SP1 has been found to be regulated by multiple miRNAs in several human cancers. In osteosarcoma, *MIR*-*493* was reported to inhibit osteosarcoma cell proliferation and invasion through targeting SP1 and therefore *MIR*-*493*/SP1 axis may be developed as potential therapeutic targets [13]. In glioblastoma multiforme, SP1 upregulation abolished the effects of *MIR*-*376a* overexpression on cell proliferation and invasion [14]. In cervical cancer, *MIR*-*296* functions as tumor suppressor via targeting SP1 [15]. However, how SP1 expression was regulated in CRC still need further investigations.

In this study, the expression and biological functions of *MIR*-*382* in CRC was investigated. The results showed *MIR*-*382* was significantly downregulated in CRC, and was associated with poorer 5-year overall survival. Luciferase activity reporter assay and western blot assay revealed that SP1 was a direct target of *MIR*-*382*. In addition, functional assays revealed that *MIR*-*382* inhibited cell proliferation and migration through targeting SP1.

## Methods

### Tissue collection

Colorectal cancer tissues and noncancerous tissues were obtained from 113 patients who underwent treatment between May 2010 and December 2012 at Cancer Hospital of China Medical University, Liaoning Cancer Hospital & Institute. Patients were excluded from the study if they have ever received anti-cancer treatments. These tissues were immediately frozen in liquid nitrogen and stored at − 80 °C for further usage. The study protocol was approved by the Research Ethics Committee of Cancer Hospital of China Medical University, Liaoning Cancer Hospital & Institute. Written informed consent was obtained from all participates.

### Cell culture

Human CRC cell lines HT29, SW480, SW620 and normal colon epithelial cell line FHC were purchased from American Type Culture Collection (ATCC, Manassas, VA, USA). CRC cells were cultured in RPMI-1640 medium (Invitrogen, Carlsbad, CA, USA) supplemented with 10% fetal bovine serum (FBS, Gibco, Gaithersburg, MD, USA). FHC cells were incubated in DMEM (Invitrogen) supplemented with 10% FBS (Gibco). These cells were maintained in a humidified atmosphere containing 5% CO_2_ at 37 °C.

### Cell transfection

*MIR*-*382* mimics, *MIR*-*382* inhibitor and the corresponding negative control (NC) were obtained from GenePharma (Shanghai, China). *SP1* siRNA (*si*-*SP1*) and NC was also obtained from GenePharma (Shanghai). To conduct cell transfection, cells were firstly cultured to about 70–90% confluence. Then, Lipofectamine 2000 (Invitrogen) was used to transfect the synthetic oligonucleotides (50 nM for miRNAs and siRNAs) according to the manufacturer’s instructions.

### Reverse transcription-quantitative polymerase chain reaction (qRT-PCR)

Total RNA was isolated from tissues and cell lines using TRIzol Reagent (Invitrogen) and was reverse transcribed to cDNA using PrimeScript RT reagent kit (Promega, Madison, WI, USA) according to the manufacturer’s instructions. qRT-PCR was conducted using SYBR Premix EX Taq™ (TaKaRa, Dalian, China) at ABI 7500 system (Applied Biosystems, Foster City, USA). *U6 snRNA* and *GAPDH* was used as endogenous controls for *MIR*-*382* and *SP1* respectively. Expression levels were measured with the relative quantification (2^−ΔΔCt^) method. The primers for *MIR*-*382*, forward 5′-CTGCAATCATTCACGGACAAC-3′ and reverse 5′-GTGTCGTCGAGTCGGCAATTC-3′; *U6 snRNA*, forward 5′-ATTGGAACGATACAGAGAAGATT-3′ and reverse 5′-GGAACGCTTCACGAATTTG-3′; *SP1*, forward 5′-TGGTGGGCAGTATGTTGT-3′ and reverse 5′-GCTATTGGCATTGGTGAA-3′; and *GAPDH*, forward 5′-ATGTCGTGGAGTCTACTGGC-3′ and reverse 5′-TGACCTTGCCCACAGCCTTG-3′.

### Western blot

Total protein was obtained from tissue and cells using RIPA lysis buffer supplemented with protease inhibitors (Beyotime, Haimen, Jiangsu, China). Protein concentration was measured using Bradford protein kit (Beyotime). Same amounts of protein samples were separated using 10% SDS-PAGE and transferred onto PVDF membrane. After blocked with 5% milk for 1 h, the membranes were incubated with the following antibodies: anti-SP1 rabbit antibody (#9389, Cell signaling Technology, Danvers, MA, USA); anti-E-Cadherin rabbit antibody (#3195, Cell signaling Technology); anti-N-Cadherin rabbit antibody (#13116, Cell signaling Technology); anti-Vimentin rabbit antibody (#5741, Cell signaling Technology); anti-Snail rabbit antibody (#3879, Cell signaling Technology); anti-GAPDH rabbit antibody (#5174, Cell signaling Technology). Signals were detected by with horseradish peroxidase-conjugated anti-rabbit IgG (#7074, Cell Signaling Technology) and enhanced chemiluminescence kit (Beyotime). Band density was analyzed using ImageJ software (NIH, Bethesda, MD, USA).

### Cell proliferation assay

Cell proliferation was determined by CCK-8 kit (Beyotime). Cells were seeded at a density of 3000 cells/well onto a 96-well plate and cultured for 0, 24, 48 and 72 h at 37 °C. At these above-mentioned points, 10 µl CCK-8 solution was added to each well and incubated for another 2 h. Optical density was then measured at 450 nm.

### Cell migration assay

Wound healing assay was conducted to measure cell migration ability. 200 μl pipette tip was used to create wound at cell surface. Photographs were taken at 0 and 48 h after wound was created and wound closure rate was measured based on these photographs using the ImageJ software (NIH).

### Luciferase activity reporter assay

Bioinformatics analysis TargetScan (http://www.targetscan.org/) was used to predict target genes of *MIR*-*382*. The wild-type 3′-UTR of *SP1* and a mutant sequence of *SP1* 3′-UTR was sub-cloned into pMIR-REPORT vector (Promega). To measure luciferase activity, cells were transfected with pMIR-SP1-3′-UTR Wt or pMIR-SP1-3′-UTR Mut, together with *MIR*-*382* mimic or NC using Lipofectamine 2000. After 48 h of transfection, cells were harvested to measure luciferase activities using dual-luciferase reporter assay system (Promega) according to the manufacturer’s protocol.

### Statistical analysis

Data were presented as the mean ± standard deviation. Differences were analyzed with two-tailed Student’s t-test (two groups) or one-way analysis of variance and Tukey test (three or above groups) using SPSS 19.0 software (IBM Corp., Armonk, NY, USA). Kaplan–Meier curve and log-rank test was used to investigate effect of *MIR*-*382* expression on overall survival. Associations between *MIR*-*382* expression and clinicopathological features were analyzed by Chi square test. Correlation between *MIR*-*382* and SP1 expression was analyzed using Person’s correlation analysis. P < 0.05 was considered as statistically significant difference.

## Results

### *MIR*-*382* was downregulated in CRC tissues and cell lines

**Fig. 1:**
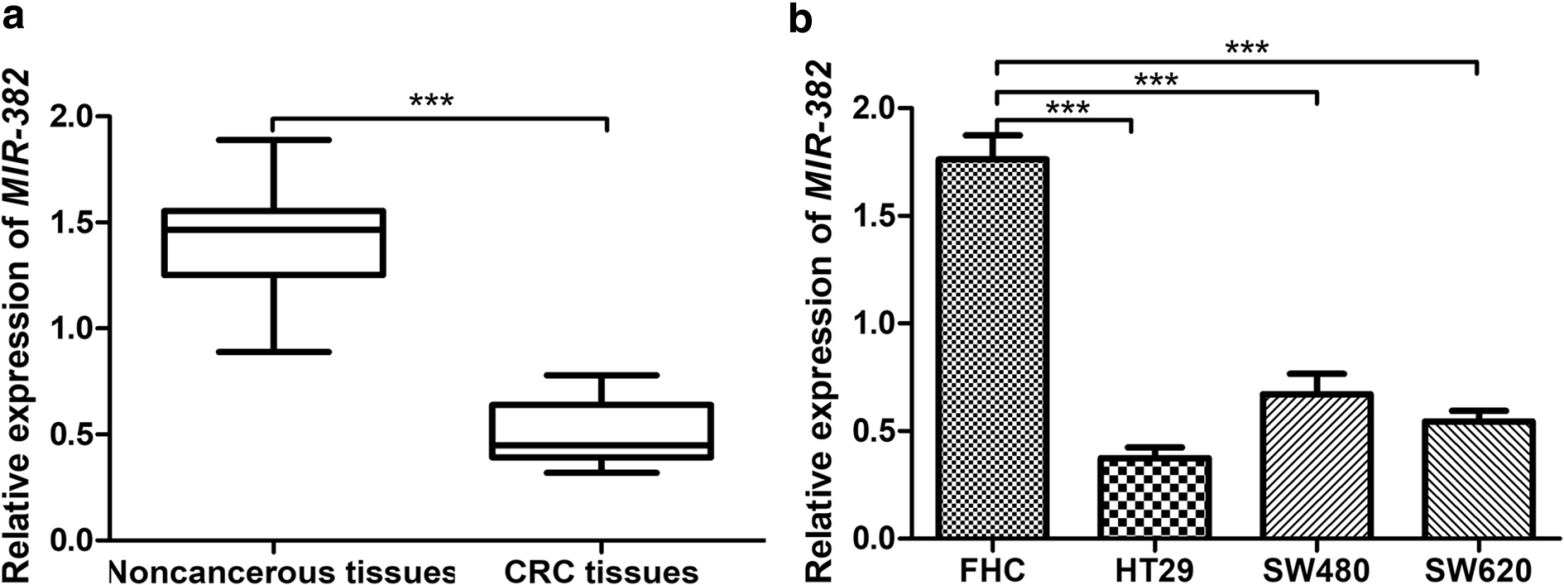
The aberrant expression of *MIR*-*382* in CRC tissues and cells. **a** The expression of *MIR*-*382* in 113 pairs of human CRC tissues and surrounding noncancerous tissues. **b** The expression of *MIR*-*382* in CRC cell lines HT29, SW480, SW620 and human normal colon epithelial cell line FHC. (***P < 0.001) *MIR*-*382*: microRNA-382; CRC: colorectal cancer

We found *MIR*-*382* expression in 113 pairs of CRC tissues was dramatically downregulated compared with corresponding noncancerous tissues using qRT-PCR (Fig. 1a). Furthermore, we measured *MIR*-*382* expression in FHC cell line and three CRC cell lines HT29, SW480, and SW620. These results showed *MIR*-*382* expression was downregulated in CRC cell lines investigated compared with FHC cell line (Fig. 1b). Besides that, we found HT29 cell line has the lowest *MIR*-*382* expression among the CRC cell lines investigated (Fig. 1b).

### Clinical significance of *MIR*-*382* expression in CRC

**Fig. 2:**
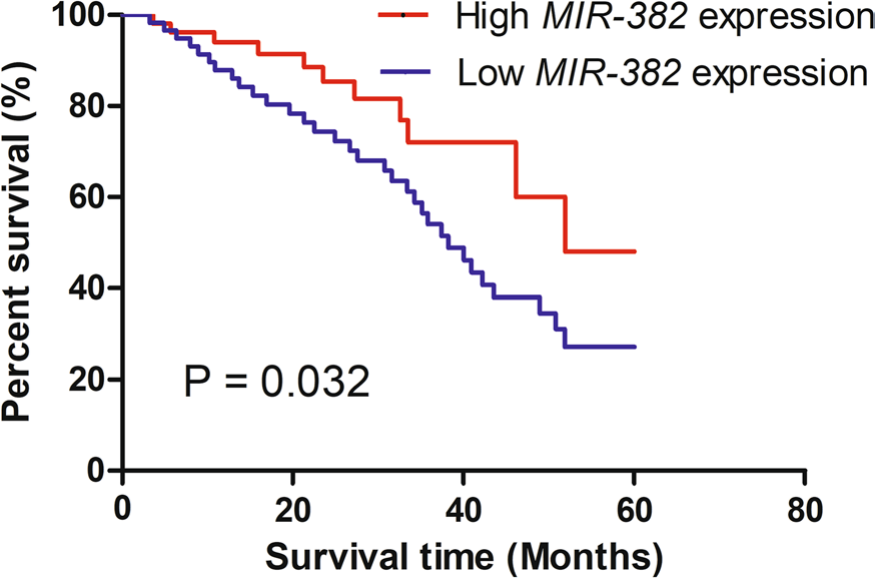
CRC patients with low *MIR*-*382* expression had a significantly shorter 5-year overall survival than those with high *MIR*-*382* expression. *MIR*-*382*: microRNA-382; CRC: colorectal cancer

Furthermore, the median of *MIR*-*382* levels was used to classify these patients into two groups: low *MIR*-*382* expression group (n = 59) and high *MIR*-*382* expression group (n = 54). Kaplan–Meier curve and log-rank test was performed to explore the associations between *MIR*-*382* expression and overall survival of CRC patients, we found low *MIR*-*382* expression predicts poorer 5-year overall survival of CRC patients (Fig. 2). We also found low *MIR*-*382* expression was strongly correlated with Lymph node metastasis and TNM stage through analyzing the associations between *MIR*-*382* and clinicopathological features (Table 1).

**Table 1:** Correlations of *MIR*-*382* expression and the clinicopathological features of CRC

Variables	No.	*MIR*-*382* expression		P value*
		Low (n = 59)	High (n = 54)	
Age (years)				
≥ 50	55	29	26	0.675
< 50	58	30	28	
Gender				
Male	50	27	23	0.092
Female	63	32	31	
Lymph node metastasis				
Absent	49	23	26	0.028
Present	64	36	28	
TNM stage				
I–II	47	25	22	0.019
III	66	34	32	

*MIR*-*382*: microRNA-382; CRC: colorectal cancer; TNM: tumor node metastasis

*Chi-square test

### *MIR*-*382* overexpression inhibits CRC cell proliferation and migration

**Fig. 3:**
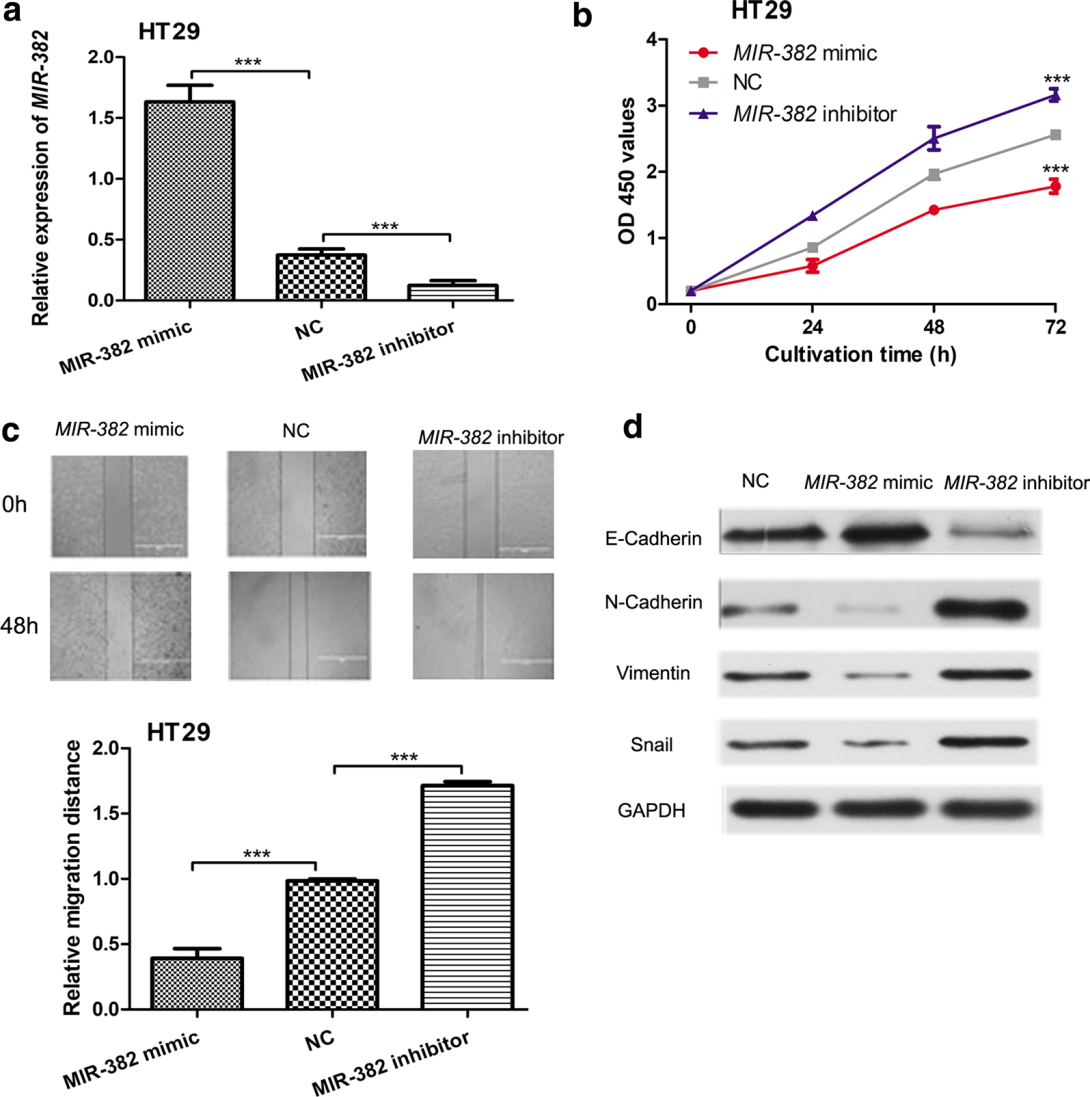
Overexpression of *MIR*-*382* inhibits cell proliferation and migration. **a** The expression of *MIR*-*382* in HT29 cell lines with synthetic miRNAs transfection. **b** CCK-8 assay was performed to measure cell proliferation in HT29 cell line after synthetic miRNAs transfection. **c** Wound-healing assay was performed to measure cell migration in HT29 cell line after synthetic miRNAs transfection. **d** Western blot was conducted to measure the expression of E-Cadherin, N-Cadherin, Vimentin, and Snail. (***P < 0.001) *MIR*-*382*: microRNA-382; NC: negative control; CCK-8: cell counting kit-8; OD 450: optical density at 450 nm

In order to investigate the effects of *MIR*-*382* on CRC cell behaviors, we transfected synthetic miRNAs into HT29 cell line. qRT-PCR results showed that *MIR*-*382* was overexpressed in *MIR*-*382* mimic transfected cells compared with NC transfected cells (Fig. 3a). CCK-8 assay showed upregulation of *MIR*-*382* inhibited HT29 cell proliferation (Fig. 3b). Wound-healing assay showed migration ability of HT29 cells transfected with *MIR*-*382* mimic was downregulated compared with those transfected with NC (Fig. 3c). We also investigated the expression of four epithelial to mesenchymal transition (EMT) markers in synthetic miRNAs transfected cell line. We found E-Cadherin was significantly downregulated; however, the expression of N-Cadherin, Vimentin, and Snail was remarkedly upregulated in *MIR*-*382* inhibitor transfected cells (Fig. 3d). However, E-Cadherin expression was upregulated but the N-Cadherin, Vimentin, and Snail expression was downregulated at cells transfected with *MIR*-*382* mimic (Fig. 3d). These results demonstrated that *MIR*-*382* functions as tumor suppressor in CRC.

### SP1 was a target of *MIR*-*382* in CRC

**Fig. 4:**
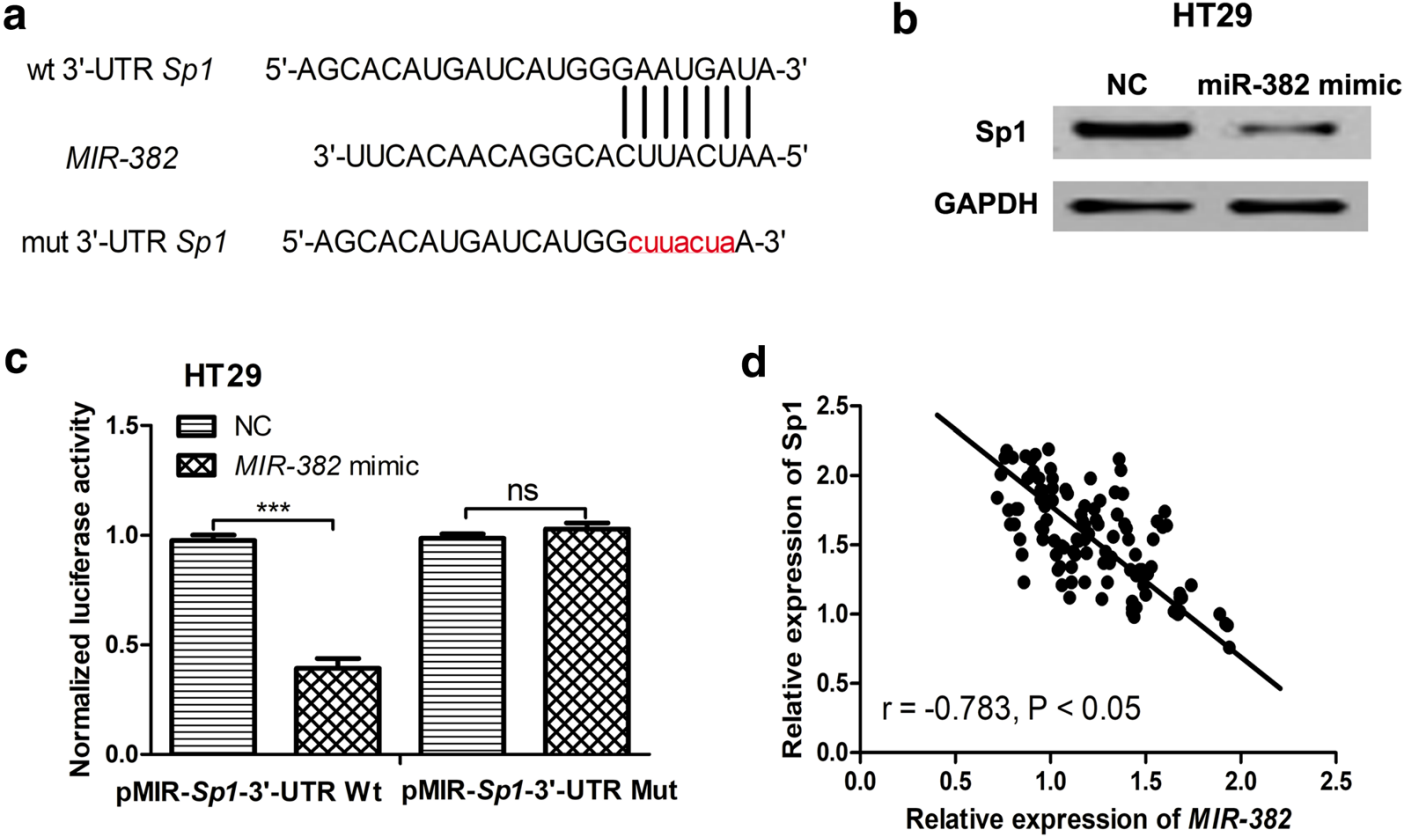
*MIR*-*382* directly target SP1 in CRC. **a** The predicted *MIR*-*382* binding site within Sp2 3′-UTR and its mutated version. **b** *MIR*-*382* mimic inhibited the expression of SP1 in HT29 cells. **c** *MIR*-*382* mimic inhibited luciferase activity of cells transfected with pMIR-SP1-3′-UTR Wt but not pMIR-SP1-3′-UTR Mut. **d** Correlation between *MIR*-*382* and SP1 expression in CRC tissues. (***P < 0.001, ns: not significant) *MIR*-*382*: microRNA-382; NC: negative control; SP1: specific protein 1; Wt: wild-type; mut: mutant; CRC: colorectal cancer; UTR: untranslated region

The results of TargetScan demonstrated that SP1 was a potential target of *MIR*-*382* (Fig. 4a). Meanwhile, we found SP1 expression was significantly reduced in HT29 cells with *MIR*-*382* mimic transfection (Fig. 4b). Subsequently, luciferase activity reporter assay was conducted to validate the connection between *MIR*-*382* and SP1. It was found that *MIR*-*382* mimic inhibited the luciferase activity of cells transfected with pMIR-SP1-3′-UTR Wt but not pMIR-SP1-3′-UTR Mut (Fig. 4c). Finally, we found an inversely correlation between *MIR*-*382* and SP1 in CRC tissues (Fig. 4d). These results revealed that SP1 was a direct target of *MIR*-*382*.

### SP1 knockdown reduces the suppressive effects of *MIR*-*382* on CRC cells

**Fig. 5:**
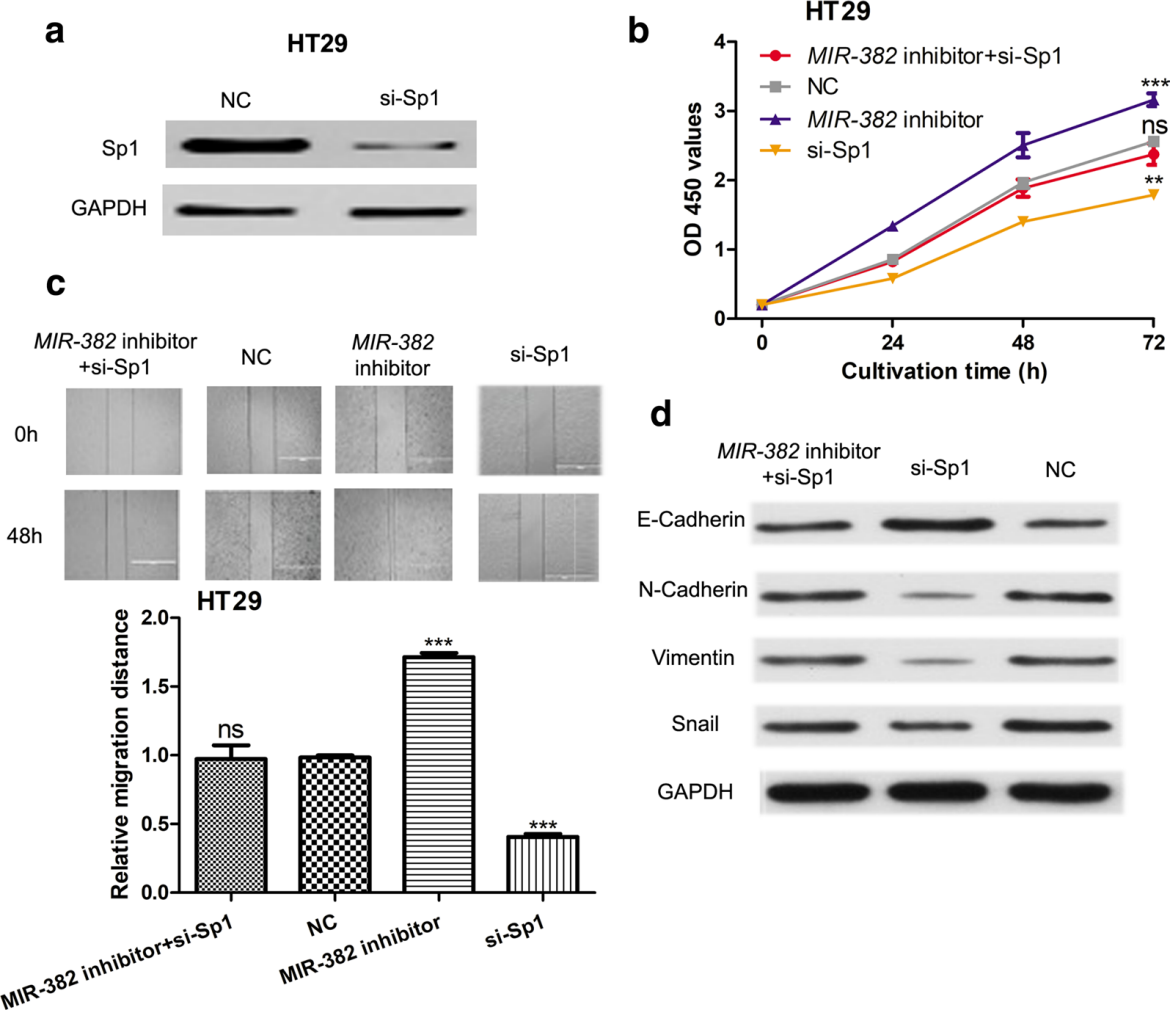
Knockdown of SP1 inhibits cell proliferation and migration. **a** The expression of SP1 in HT29 cell lines with synthetic siRNAs transfection. **b** CCK-8 assay was performed to measure cell proliferation in HT29 cell line with synthetic siRNAs or miRNAs transfection. **c** Wound-healing assay was performed to measure cell migration in HT29 cell line with synthetic siRNAs or miRNAs transfection. **d** Western blot was conducted to measure the expression of E-Cadherin, N-Cadherin, Vimentin, and Snail. (***P < 0.001, **P < 0.01, ns: not significant) *MIR*-*382*: microRNA-382; NC: negative control; SP1: specific protein 1; si-SP1: siRNA targeting SP1; CCK-8: cell counting kit-8; OD 450: optical density at 450 nm

To determine the critical mediator of *MIR*-*382* induced effects on cell proliferation and migration, loss-of-function experiment was conducted. We found *si*-*SP1* transfection significantly reduced SP1 expression in HT29 cells (Fig. 5a). As shown in Fig. 5b, the knockdown of SP1 partially abrogated the stimulation effect of *MIR*-*382* inhibitor on cell proliferation. Wound-healing assay showed that *si*-*SP1* abolished the stimulation effects of *MIR*-*382* inhibitor on cell migration (Fig. 5c). In the meantime, we found knockdown of SP1 increased E-Cadherin expression but decreased the expression of N-Cadherin, Vimentin, and Snail (Fig. 5d).

## Discussion

Extensive efforts to develop cancer treatment methods have been made but cancer is still a global health burden [1, 16, 17]. Emerging evidence showed miRNAs were deeply participated in human cancers [18]. *MIR*-*382* was found act as tumor suppressor and involved in the pathogenesis of various human cancers including hepatocellular carcinoma, ovarian cancer, and osteosarcoma [19–21]. For instance, Zhang et al. reported *MIR*-*382* is able to inhibit hepatocellular carcinoma metastasis by targeting Golgi Membrane Protein 1 [19]. Also, they reported low *MIR*-*382* expression is an independent prognostic factor for hepatocellular carcinoma [19]. Moreover, Tan et al. found *MIR*-*382* could inhibit ovarian cancer cell proliferation, migration, invasion and epithelial-mesenchymal transition through regulating receptor tyrosine kinase orphan receptor 1 [20]. However, the precise mechanism of *MIR*-*382* in regulating CRC progression remains need further investigations.

In this study, we examined *MIR*-*382* expression in CRC and we found *MIR*-*382* expression was significantly reduced in CRC tissues and cell lines. Furthermore, we found low *MIR*-*382* expression indicated poorer 5-year overall survival of CRC patients. Given *MIR*-*382* expression was downregulated in CRC tissues and cell lines, we therefore interested to investigate the effects of *MIR*-*382* on CRC cell proliferation and migration. Functional assays demonstrated that *MIR*-*382* overexpression inhibits cell proliferation and migration in vitro. EMT is considered as key step for invasion and metastasis [22]. We showed *MIR*-*382* overexpression decreased the expression of mesenchymal markers including N-Cadherin, Vimentin, and Snail but increased the expression of epithelial marker E-Cadherin. These results revealed that *MIR*-*382* functions as a tumor suppressor in CRC.

The above results provided us a preliminarily understanding of the critical role of *MIR*-*382* in CRC, however, the precise mechanism behind these effects were still unknown. By utilizing bioinformatic analysis and western blot, we found SP1 was a direct target of *MIR*-*382*. Interestingly, SP1 was previously demonstrated to be overexpressed in CRC and correlated with CRC metastasis [10]. Also, it was established that SP1 is involved in the growth and progression of tumors [5, 10]. Therefore, it is interesting to investigate whether SP1 was a mediator for the inhibitory role of *MIR*-*382* in CRC. Our results demonstrated that the knockdown of SP1 abolished the effects of *MIR*-*382* on cell proliferation and migration. We also searched the validated targets of *MIR*-*382* in miRTarBase (http://mirtarbase.mbc.nctu.edu.tw/php/index.php) and found SP1 was not included in the current version database. Therefore, our study may provide evidence that SP1 was a novel target for *MIR*-*382*.

## Conclusions

In conclusions, our results demonstrated that *MIR*-*382* acts as a tumor suppressive miRNA in CRC progression. *MIR*-*382* inhibited CRC proliferation and migration through targeting SP1. Taken together, these findings highlighted the importance of *MIR*-*382*/SP1 axis in the progression of CRC and may provide novel targets for CRC treatment.

## Abbreviations

*MIR*-*382*: microRNA-382
CRC: colorectal cancer
RT-qPCR: reverse transcription-quantitative polymerase chain reaction
CCK-8: cell counting kit-8
NC: negative control
SP1: specific protein 1
Wt: wild type
Mut: mutant
UTR: untranslated region
si-SP1: small interfering RNA targeting SP1
